# Knowledge, attitude/perception, and practice related to seasonal influenza vaccination among caregivers of young Thai children: A cross-sectional study

**DOI:** 10.1371/journal.pone.0253561

**Published:** 2021-06-25

**Authors:** Chareeya Thanee, Wanitchaya Kittikraisak, Chalinthorn Sinthuwattanawibool, Koonkoaw Roekworachai, Arunee Klinklom, Katesiree Kornsitthikul, Suwadee Jirasakpisarn, Ussanee Srirompotong, Malinee Chittaganpitch, Fatimah S. Dawood, Piyarat Suntarattiwong, Joshua A. Mott, Tawee Chotpitayasunondh

**Affiliations:** 1 Sunpasitthiprasong Hospital, Ministry of Public Health, Ubon Ratchathani, Thailand; 2 Influenza Program, Thailand Ministry of Public Health–U.S. Centers for Disease Control and Prevention Collaboration, Nonthaburi, Thailand; 3 Nakornping Hospital, Ministry of Public Health, Chiang Mai, Thailand; 4 Surat Thani Hospital, Ministry of Public Health, Surat Thani, Thailand; 5 Chonburi Hospital, Ministry of Public Health, Chonburi, Thailand; 6 Pranangklao Hospital, Ministry of Public Health, Nonthaburi, Thailand; 7 Khonkaen Hospital, Ministry of Public Health, Khon Kaen, Thailand; 8 Department of Medical Sciences, Ministry of Public Health, Nonthaburi, Thailand; 9 Influenza Division, U.S. Centers for Disease Control and Prevention, Atlanta, Georgia, United States of America; 10 Queen Sirikit National Institute of Child Health, Ministry of Public Health, Bangkok, Thailand; Bucharest University of Economic Studies, ROMANIA

## Abstract

**Background:**

Seasonal influenza vaccination uptake among young children in Thailand is low despite national recommendation for vaccination. We implemented a knowledge, attitude/perception, and practice survey to understand determinants of influenza vaccination in children aged six months to two years.

**Methods:**

Using a cross-sectional design, we interviewed caregivers of 700 children in seven hospitals using a structured questionnaire to collect information on caregivers’ and children’s demographic characteristics, and caregivers’ knowledge of influenza illness and national vaccine recommendation, attitude/perception toward influenza vaccine, and information sources. We verified children’s influenza vaccination status against medical records (vaccinated vs. unvaccinated). Logistic regression was used to examine factors independently associated with children receiving influenza vaccination in the 2018 season using the dataset restricted to only children’s parents. Variables associated with vaccination at p-value ≤0.20 were included in subsequent multivariable logistic models. Significant independent determinants of children’s influenza vaccination and collinearity of covariates were assessed. The final model was constructed using a stepwise backward elimination approach with variables significant at p-value <0.05 retained in the model.

**Results:**

During August 2018-February 2019, 700 children’s caregivers completed the questionnaire; 61 (9%) were caregivers of vaccinated children. Caregivers of the vaccinated children were statistically more likely to have higher education (61% vs. 38%; p-value<0.01) and to know of influenza illness (93% vs. 76%; p-value = 0.03) than those of the unvaccinated group. Factors associated with children receiving influenza vaccination were identifying healthcare providers as a primary source of information about influenza illness for parents (adjusted odds ratio [aOR], 2.8; 95% confidence interval [CI], 1.3–6.0), parents’ strongly agreeing with the national recommendation for influenza vaccination in young children (aOR, 2.9; 95% CI, 1.5–5.9), using health insurance provided by the government or parent’s employer for children’s doctor visits (aOR, 2.6; 95% CI, 1.1–6.6), and the children’s history of receiving influenza vaccination in the 2017 season or earlier (aOR, 3.2; 95% CI, 1.4–7.8).

**Conclusion:**

The majority of caregivers of children in this study had knowledge of influenza illness and influenza vaccine. Caregivers reported various sources of information regarding influenza illness and the vaccine, but healthcare providers remained the most trusted source. Children’s history of influenza vaccination in prior season(s) was the strongest determinant of children being vaccinated for influenza in the current season.

## Introduction

Influenza viruses are members of the family Orthomyxoviridae. Influenza virus infection is an important cause of respiratory illness that results in hospitalizations and deaths among children <5 years around the world [1, 2]. A systematic review of data from the literature and surveillance platforms during 1982–2012 showed that each year influenza resulted in about 374,000 hospitalizations among children aged <1 year old (including 228,000 hospitalizations among children aged <6 months) and nearly one million hospitalizations among children aged <5 years [1]. Influenza vaccination is the most effective way to prevent influenza illness and severe complications from influenza virus infection [2–7].

In Thailand, annual influenza vaccination is recommended for persons aged ≥65 years, children aged six months to two years (i.e., <36 months), persons with chronic medical conditions, pregnant women from 2^nd^ trimester, persons weighing >100 kg and/or having a body mass index ≥35 kg/m^2^, institutionalized mentally disabled persons, and healthcare personnel. Seasonal trivalent influenza vaccination is offered free of charge to recommended groups through annual national campaigns. However, in a prospective cohort of Thai children during 2011–2013, vaccine coverage among children aged six months to two years actively followed regularly by pediatricians was only 31% [3] and an estimated nationwide coverage among young children in a more recent year (2015) was only 2% [4].

In Thailand, the parent must approve any decision to vaccinate a child. However, there are many factors that may relate to parental consent for vaccination including parental education [5], child’s age [6], parent’s income [5, 6], social norms [5–9], the cost of vaccination [5–18], parent’s knowledge about influenza virus infection and influenza vaccination [5, 6, 9], and parent’s perceptions about vaccine safety and benefits [6, 8, 9]. In studies from Korea, Singapore, and the USA, a physician’s recommendation to parents to vaccinate for influenza was the most important factor to achieve vaccination in children [5, 7, 8, 15]. However, the influence of physicians on parental decisions regarding child vaccination may be setting-specific and driven by different healthcare infrastructures and social contexts. In this study, we conducted a Knowledge, Attitude/perception, and Practice (KAP) survey among caregivers (parents, other family members, or hired nannies) of young Thai children to understand the determinants of influenza vaccination in their children.

## Methods

### Study design and population

A cross-sectional survey was conducted from August 2018 through February 2019 among caregivers of children aged six months to two years old (i.e., <36 months) seeking care for influenza-like illness. These children were enrolled by convenient sampling into a multi-site network of outpatient and inpatient departments in seven government hospitals to estimate seasonal influenza vaccine effectiveness among young children in Thailand. Site selection for the network was based on geographic location, number of children cared for per year, laboratory support in the province, and the interest of the hospital to join this network. Following written informed consent, the caregivers of the children enrolled were interviewed by trained study nurses using a structured questionnaire.

### Questionnaire

The KAP constructs were measured with multiple items that examined different aspects of the conceptual content, drawn from previously validated items [19–21]. The KAP questionnaire included 16 questions (**S1 File**) on the respondent’s demographics (such as age, education, the relationship to the child); awareness and knowledge of the Thai Ministry of Public Health’s (MOPH’s) influenza vaccine recommendation; understanding of influenza illness; perceptions toward seasonal influenza vaccine; information sources for influenza illness and influenza vaccine; trusted information sources regarding influenza vaccine; reported influenza vaccination history; and willingness to have children receive seasonal influenza vaccine. Different response scales (e.g., 5-point agree to disagree scale and other Likert-like scales) were used to optimize measured variability and minimize measurement error. After the pre-test, the questionnaire was translated into Thai language for use. Children’s actual receipt of influenza vaccine was used to indicate practice toward influenza vaccination.

### Influenza vaccine campaign in Thailand

The national influenza vaccine campaign in Thailand, which is run by the Thai MOPH, begins around May or June each year. Annually, about four million doses of the trivalent influenza vaccine are available free of charge for about three months during the campaign to all recommended risk groups (total 11 million individuals), including children (approximately 1.5 million) [22]. Vaccination is provided on a first-come, first-served basis. When the free-of-charge vaccine is used up, parents have an option to purchase the trivalent influenza vaccine for their children from other sources. Additionally, quadrivalent influenza vaccine is also available for self-purchase during and after the MOPH’s campaign in all seven hospitals participating in this study. Any decision to vaccinate a child was based on the attending physician and the parent(s) who accompanied the child to doctor visit. This study had no influence on this decision and influenza vaccination was not part of the enrollment criteria. The survey was initiated approximately three months following the annual campaign’s commencement in order to allow children to have an opportunity to be vaccinated.

### Influenza vaccination definitions and verification

A child’s influenza vaccination status was verified using a vaccine book and/or medical records. A previously unvaccinated child was considered fully vaccinated against influenza if he/she received two doses of influenza vaccine ≥28 days apart during the current season. A previously vaccinated child was considered fully vaccinated if he/she received one dose of the vaccine during the current season and received two doses of the vaccine in any prior season. A child was considered partially vaccinated against influenza if he/she received one dose of influenza vaccine during the current influenza season but had never been fully vaccinated in any prior season. A child was considered unvaccinated against influenza if he/she did not receive any influenza vaccine during the current influenza season or received the vaccine <14 days prior to illness onset.

### Sample size

Sample size was calculated based on proportion of population with a certain characteristic, influenza vaccination coverage, power of 80%, and an alpha of 0.05. Assuming a conservative vaccination coverage of 5% and a 40% prevalence of each characteristic, a sample size of 400 would allow us to detect an odds ratio of 3.5 of receiving influenza vaccination among those having a certain characteristic vs. those who do not. To account for increased correlation within hospital catchments (design effort of 1.5) and missing information, a total sample size of 700 was needed for this KAP survey.

### Statistical analysis

All analyses were conducted using Stata version 16 (StataCorp, College Station, Texas). Only respondents who knew about influenza were included in the knowledge and attitude/perception analyses. Characteristics of vaccinated and unvaccinated children and their caregivers who responded to the survey were compared using the Chi-square test or Fisher’s exact test as appropriate. Proportions were calculated to evaluate awareness, knowledge, attitude/perception toward seasonal influenza vaccine, and information sources in univariate analyses. For the purpose of the analysis, responses to the questions on awareness, knowledge, and attitude/perception were coded into categories depending on the distribution of the responses. For example, the respondents’ answers to the question regarding the MOPH’s recommendation for influenza vaccination in young children were coded into “answered correctly”, “answered incorrectly, and “unaware of the recommendation”. Respondents’ attitude/perception toward the harmfulness of seasonal influenza virus infection were coded into “very worried”, “somewhat worried”, “not too worried”, “not at all worried”, and “refused to answer”. Similarly, responses to question related to attitude/perception about influenza vaccine safety in young children were coded into “completely safe”, “very safe”, “somewhat safe”, “somewhat unsafe”, “completely unsafe”, and “not sure”.

For the analyses of factors associated with influenza vaccination in children, the dataset was further restricted to include only parents who knew about seasonal influenza vaccine. Respondents who were not parents were excluded because they had no authority to make any decision in terms of the children’s vaccination. For these analyses, characteristics of vaccinated and unvaccinated children as well as those of the respondents (with some variables collapsed to avoid zero cells) were compared using bivariate logistic regression for which the dependent variable was child’s vaccination (yes or no) in the 2018 influenza season. Variables associated with vaccination at p-value ≤0.20 were included in subsequent multivariable logistic models. Significant independent determinants of children’s influenza vaccination and collinearity of covariates were assessed. The final model was constructed using a stepwise backward elimination approach with variables significant at p-value <0.05 retained in the model. Two-tailed p-values of <0.05 were considered statistically significant.

### Ethical approval

This study was approved by ethics committees of all participating hospitals (Sunpasitthiprasong Hospital, Nakornping Hospital, Surat Thani Hospital, Chonburi Hospital, Pranangklao Hospital, Khon Kaen Hospital, and Queen Sirikit National Institute of Child Health) and the U.S. Centers for Disease Control and Prevention’s Institutional Review Board. All respondents provided written informed consent to participate.

## Results

### Demographic characteristics of respondents and children

During August 2018-February 2019, 700 respondents completed the KAP survey. Sixty-one (9%) were caregivers of vaccinated children and 639 (91%) were caregivers of unvaccinated children. Generally, characteristics of the caregivers of vaccinated and unvaccinated children were similar in terms of relationship to the enrolled children, age, sex, knowledge about influenza vaccine, and whether the caregivers themselves had ever been vaccinated with influenza vaccine **(Table 1)**. However, vaccinated children were statistically more likely to have caregivers with higher education and to know of influenza as a disease than unvaccinated children. Specifically, a higher proportion of caregivers of vaccinated children completed at least a diploma or higher vocational school compared those of unvaccinated children (61% vs. 38%; p-value<0.01). Nearly all caregivers of vaccinated children knew of influenza illness while only about three fourths of unvaccinated children reported so (93% vs. 76%; p-value = 0.03).

**Table 1:** Characteristics of respondents and children who completed the knowledge, attitude/perception, and practice survey, Bangkok, Thailand 2018.

	All children (N = 700) n (%)	Vaccinated children^a^ (N = 61) n (%)	Unvaccinated children^a^ (N = 639) n (%)	p-value
**ENROLLMENT SETTING**				
**Study site and location**				0.06
Queen Sirikit National Institute of Child Health, Bangkok	100 (14)	8 (13)	92 (14)	
Chonburi Hospital, Chonburi	100 (14)	8 (13)	92 (14)	
Khon Kaen Hospital, Khon Kaen	100 (14)	5 (8)	95 (15)	
Nakornping Hospital, Chiang Mai	100 (14)	16 (26)	84 (13)	
Pranangklao Hospital, Nonthaburi	100 (14)	4 (7)	96 (15)	
Sunpasitthiprasong Hospital, Ubonratchathani	100 (14)	9 (15)	91 (14)	
Surat Thani Hospital, Surat Thani	100 (14)	11 (18)	89 (14)	
**Department**				0.93
Outpatient	629 (90)	55 (90)	574 (90)	
Inpatient	71 (10)	6 (10)	65 (10)	
**RESPONDENTS’ CHARACTERISTICS**				
**Relationship with the enrolled child**				0.58
Parent	627 (90)	57 (93)	570 (89)	
Other family member	61 (9)	3 (5)	58 (9)	
Hired nanny	5 (<1)	0 (0)	5 (1)	
Other	7 (1)	1 (2)	6 (1)	
**Age at enrollment (years)**				0.46
<20	32 (5)	3 (5)	29 (5)	
20–29	318 (45)	24 (39)	294 (46)	
30–39	243 (35)	28 (46)	215 (34)	
40–49	62 (9)	4 (7)	58 (9)	
50–59	36 (5)	2 (3)	34 (95)	
≥60	9 (1)	0 (0)	0 (0)	
**Sex**				0.52
Female	560 (80)	52 (85)	508 (79)	
Male	67 (10)	5 (8)	62 (10)	
**Highest education**				<0.01
At least diploma/higher vocational school	272 (39)	37 (61)	235 (38)	
Lower than diploma or higher vocational school	404 (58)	24 (39)	380 (59)	
No schooling	24 (3)	0 (0)	24 (4)	
**Knew of influenza illness**				0.03
Yes	541 (77)	57 (93)	484 (76)	
No	132 (19)	2 (3)	130 (20)	
Not sure or refused to answer	27 (4)	2 (3)	25 (4)	
**Knew of influenza vaccine**				0.07
Yes	469 (87)	55 (96)	414 (86)	
No	49 (9)	1 (2)	48 (10)	
Not sure	23 (4)	1 (2)	22 (4)	
**Ever had seasonal influenza vaccination**^**b**^				0.61
Yes	57 (8)	6 (10)	51 (8)	
No	643 (92)	55 (90)	588 (92)	
**CHILDREN’S CHARACTERISTICS**				
**Age at enrollment (months)**				0.40
6 to <12	207 (30)	193 (30)	14 (23)	
12 to <24	304 (43)	277 (43)	27 (44)	
24 to <36	189 (27)	169 (26)	20 (33)	
**Sex**				0.95
Female	301 (43)	26 (43)	275 (43)	
Male	399 (57)	35 (57)	364 (57)	
**Had existing medical condition(s)**^**b**^				0.27
No	586 (84)	48 (79)	538 (84)	
1 condition	86 (12)	9 (15)	77 (12)	
≥2 conditions	28 (4)	4 (7)	24 (4)	
**Health insurance coverage during the enrollment visit**				0.03
Universal coverage	536 (77)	47 (77)	489 (77)	
Civil service	32 (5)	7 (11)	25 (4)	
Private	2 (<1)	0 (0)	2 (<1)	
Out-of-pocket	130 (19)	7 (11)	123 (19)	
**Lived in household with smoking family member(s)**				0.03
Yes	375 (54)	23 (38)	352 (55)	
No	324 (46)	38 (62)	286 (45)	
Not sure	1 (<1)	0 (0)	1 (<1)	
**Received influenza vaccination in the 2017 influenza season or earlier**				<0.01
No	667 (95)	48 (79)	619 (97)	
1 dose	10 (1)	4 (7)	6 (1)	
≥2 doses	23 (3)	9 (15)	14 (2)	

^a^During the influenza season when the survey was conducted (2018).

^b^Reported by respondents.

The vaccinated and unvaccinated children were similar in terms of age, sex, and presence of an existing medical condition **(Table 1)**. However, they differed significantly by how they paid for their doctor visits, whether or not they lived in a household with smoking family member(s), and influenza vaccination history in the 2017 influenza season or earlier. Specifically, a higher proportion of unvaccinated children reported paying out-of-pocket for doctor visits than their vaccinated counterparts (19% vs. 11%; p-value = 0.04). In addition, a higher proportion of unvaccinated children lived in a household with smoking family member(s) compared to those vaccinated (55% vs. 38%; p-value = 0.02).

All enrolled children had influenza vaccination status verified. Among 61 children who received influenza vaccination in the 2018 season (9% of 700; the coverage ranged from 4–16% across seven sites), 28 (46%) were fully vaccinated and 33 (54%) were partially vaccinated. Among the 28 fully vaccinated children, 11 (39%) received trivalent influenza vaccine, 3 (11%) received both trivalent and quadrivalent vaccines, 10 (36%) received only quadrivalent influenza vaccine, and 4 (14%) had no record of vaccine type.

### Information source about influenza illness and influenza vaccine

**Fig 1:**
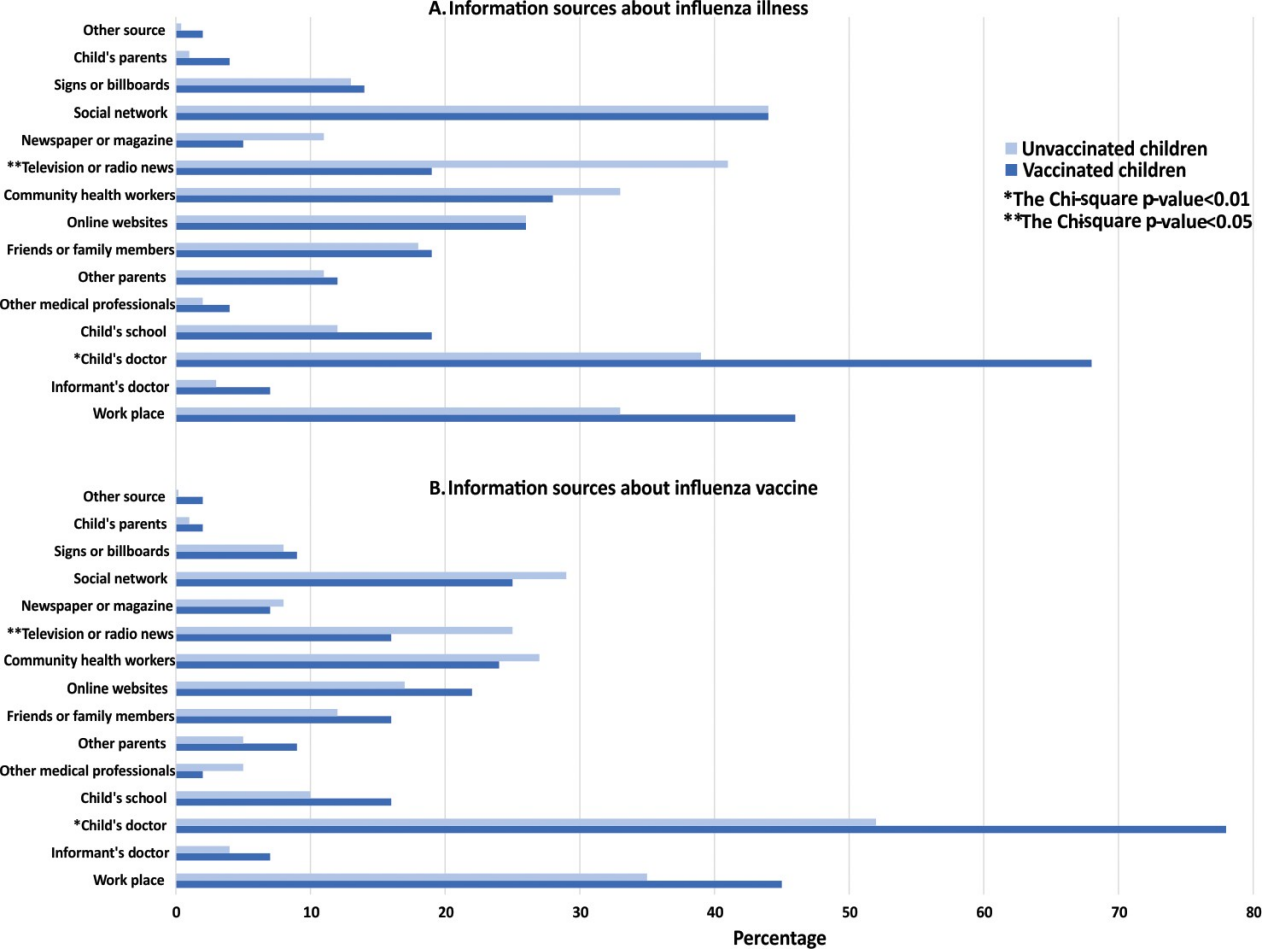
Information sources about influenza illness and influenza vaccine for children. For source of information regarding influenza, only those who knew of influenza were included and for source of information regarding influenza vaccine, only those who knew of influenza vaccine were included. All sources that apply could be selected.

**Fig 2:**
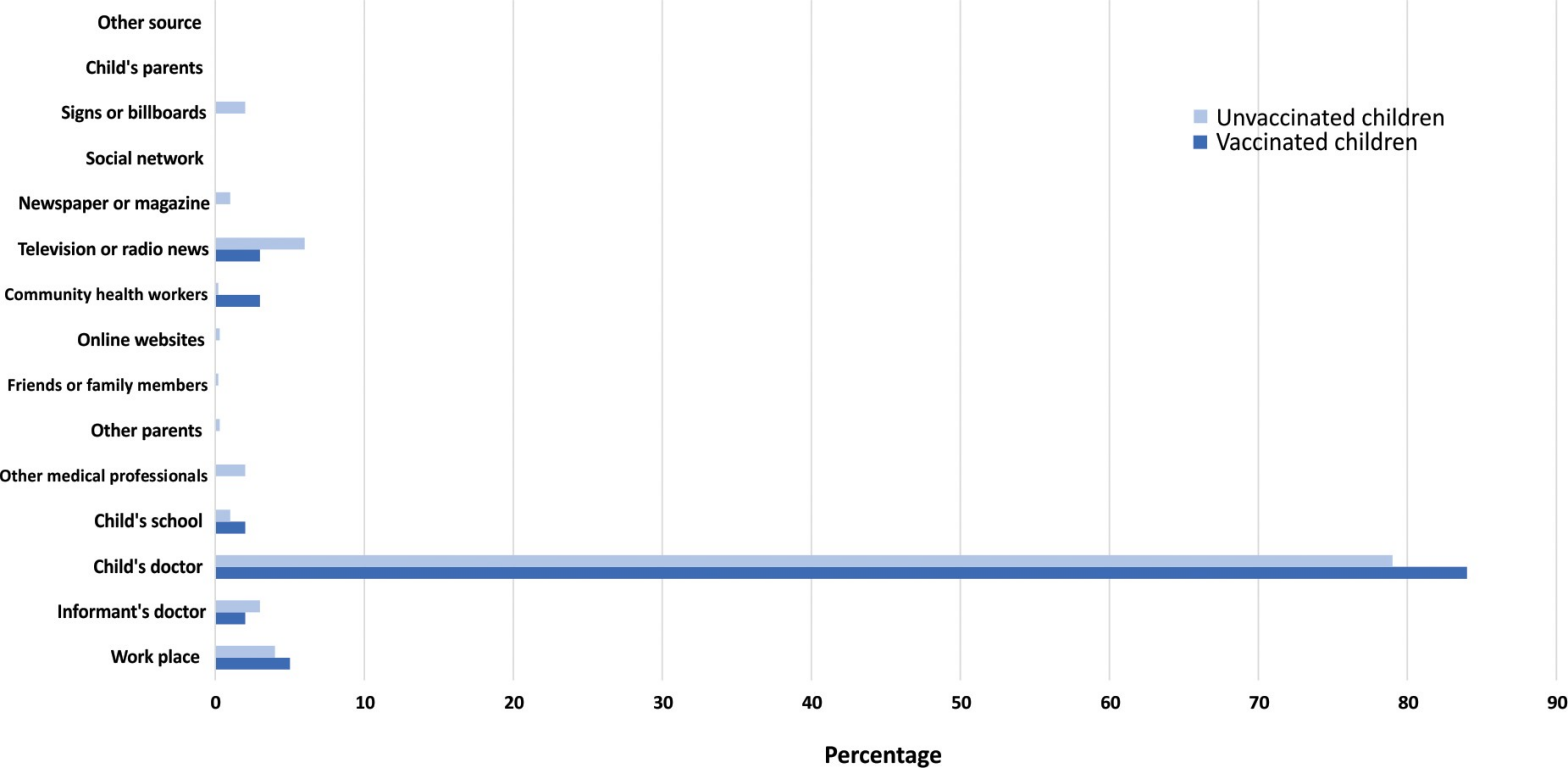
The most trusted source about influenza vaccine for children.

Of 700 respondents, 541 (77%) reported having heard of influenza illness prior to study enrollment. Of these, 469 (87%) also knew of the influenza vaccine. Respondents who knew of influenza illness and influenza vaccine (n = 469) received information about influenza illness and the vaccine from multiple sources **(Fig 1A and 1B)**. The most common source for influenza illness information was social network (44%), followed by the child’s doctor (43%), television or radio news (39%), and respondent’s workplace (34%). The most common source of information regarding influenza vaccine was the child’s doctor (55%), followed by respondent’s workplace (36%), social network (28%), and community health workers (27%). However, the most trusted source regarding influenza vaccine for children was the child’s doctor (80%; **Fig 2**).

### Attitude/perception toward influenza illness and influenza vaccine

Among 541 respondents who knew about influenza illness, similar proportions of vaccinated and unvaccinated children had caregivers who were worried about the child getting sick with influenza **(Table 2).** Their perceived likelihood of the enrolled children being harmed by seasonal influenza virus infection was also not significantly different.

**Table 2:** Respondents’ information source, knowledge, attitude/perception toward influenza illness and influenza vaccine in the 2018 influenza season in children aged six months to two years^a^.

	All children N = 541	Vaccinated children (N = 57) n (%)	Unvaccinated children (N = 484) n (%)	p-value
**Information source about influenza illness**				<0.01
Healthcare providers	333 (62)	48 (84)	285 (59)	
Others	208 (38)	9 (16)	199 (41)	
**Worried about the enrolled child getting sick with seasonal influenza virus infection during the current influenza season**				0.95
Very worried	239 (44)	25 (44)	214 (44)	
Somewhat worried	264 (49)	28 (49)	236 (49)	
Not too worried	33 (6)	4 (7)	29 (6)	
Not at all worried	4 (1)	0 (0)	4 (1)	
Refused to answer	1 (<1)	0 (0)	1 (<1)	
**Perceived likelihood of the enrolled child being harm by seasonal influenza virus infection**				0.29
Very likely	177 (33)	18 (32)	159 (33)	
Somewhat likely	230 (43)	24 (42)	206 (43)	
Somewhat unlikely	126 (23)	13 (23)	113 (23)	
Very unlikely	3 (<1)	0 (0)	3 (1)	
Not sure	5 (1)	2 (4)	3 (1)	
**Information source about influenza vaccine**^**b**^				<0.01
Healthcare providers	330 (70)	47 (85)	283 (68)	
Others	139 (30)	8 (15)	131 (32)	
**Most trusted information source about influenza vaccine**^**b**^				0.47
Child’s doctor	375 (80)	46 (84)	329 (79)	
Other	94 (20)	9 (16)	85 (21)	
**Knowledge of Ministry of Public Health’s recommendation for influenza vaccination in young children**^**b**^				0.02
Answered correctly	380 (81)	52 (95)	328 (79)	
Answered incorrectly	2 (<1)	0 (0)	2 (<1)	
Unaware of the recommendation	87 (16)	3 (5)	84 (20)	
**Agreement with Ministry of Public Health’s recommendation for influenza vaccination in young children**^**b**^				<0.01
Strongly agreed	267 (57)	42 (76)	225 (54)	
Agreed	198 (42)	13 (24)	185 (45)	
Disagreed	0 (0)	0 (0)	0 (0)	
Strongly disagreed	0 (0)	0 (0)	0 (0)	
Not sure	4 (1)	0 (0)	4 (1)	
**Perceptions about influenza vaccine effectiveness among young children**^**b**^				0.31
Very effective	178 (38)	24 (44)	154 (37)	
Somewhat effective	268 (57)	31 (56)	237 (57)	
Not too effective	5 (1)	0 (0)	5 (1)	
Not sure	18 (4)	0 (0)	18 (4)	
**Perceptions about influenza vaccine safety in young children**^**b**^				0.02
Completely safe	133 (28)	24 (44)	109 (26)	
Very safe	197 (42)	22 (40)	175 (42)	
Somewhat safe	126 (27	9 (16)	117 (28)	
Somewhat unsafe	0 (0)	0 (0)	0 (0)	
Completely unsafe	0 (0)	0 (0)	0 (0)	
Not sure	13 (3)	0 (0)	13 (3)	
**Intention for the enrolled child to get vaccinated if offered**^**b**^				0.48
Yes	459 (98)	53 (96)	406 (98)	
No	3 (1)	0 (0)	3 (1)	
Not sure	4 (1)	1 (2)	3 (1)	
Refused to answer	3 (1)	1 (2)	2 (<1)	

^a^The analytic dataset was restricted to those who knew of influenza illness prior to study enrollment.

^b^The dataset was further restricted to include only those who knew of influenza vaccine in variables related to the vaccine.

Of 469 respondents who knew of influenza vaccine, most were aware of the MOPH’s recommendation for influenza vaccination in children, and this differed by vaccination status (95% in the vaccinated group vs. 79% in the unvaccinated group; p-value = 0.02). More caregivers of vaccinated children strongly agreed with the recommendation compared to those of unvaccinated children (76% vs. 54%; p-value<0.01). Both groups of caregivers perceived the vaccine as very effective or somewhat effective. However, attitude/perception toward influenza vaccine safety among young children was significantly different. Specifically, 44% of the caregivers of vaccinated children perceived the vaccine as completely safe compared to 26% of the caregivers of unvaccinated children (p-value 0.02). Lastly, intention to have the enrolled children vaccinated was similar in both groups of caregivers.

### Factors associated with children receiving influenza vaccination in the 2018 season

Using the dataset restricted to include only parents who could make vaccination decisions for their children, factors independently associated with children having received influenza vaccination in the 2018 season included parents identifying healthcare providers as an information source for influenza illness (adjusted odds ratio [aOR], 2.8; 95% confidence interval [CI], 1.3–6.0; **Table 3**), parents’ agreement with the MOPH’s recommendation for influenza vaccination in young children (aOR, 2.9; 95% CI, 1.5–5.9), and the children’s prior vaccination in the 2017 season or earlier (aOR, 3.2; 95% CI, 1.4–7.8). Children were more likely to receive influenza vaccination when they used health insurance provided by the government or parent’s employer to pay for the children’s doctor visits (aOR, 2.6; 95% CI, 1.1–6.6).

**Table 3:** Factors associated with children having received influenza vaccination in the 2018 influenza season^a^.

	Total children	Vaccinated children N (%)	Odds ratio (95% CI)	Adjusted odds ratio (95% CI)
**Study site and location**				
Queen Sirikit National Institute of Child Health, Bangkok	85	8 (9)	Reference	
Chonburi Hospital, Chonburi	78	8 (10)	1.1 (0.4–3.1)	
Khon Kaen Hospital, Khon Kaen	33	4 (12)	1.3 (0.4–4.7)	
Nakornping Hospital, Chiang Mai	45	14 (31)	4.3 (1.7–11.4)	
Pranangklao Hospital, Nonthaburi	44	4 (9)	1.0 (0.3–3.4)	
Sunpasitthiprasong Hospital, Ubonratchathani	54	4 (7)	0.8 (0.2–2.7)	
Surat Thani Hospital, Surat Thani	100	11 (11)	1.2 (0.5–3.1)	
**Parent’s highest education**				
At least diploma/higher vocational school	211	34 (16)	Reference	
Lower than diploma or higher vocational school	228	19 (8)	2.1 (1.2–3.8)	
**Parents’ information source about influenza illness**				
Healthcare providers	283	44 (16)	3.0 (1.4–6.3)	2.8 (1.3–6.0)
Others	156	9 (6)	Reference	
**Parent’s knowledge of Ministry of Public Health’s recommendation for influenza vaccination in young children**				
Answered correctly	360	50 (14)	4.1 (1.2–13.5)	
Answered incorrectly or unaware of the recommendation	79	3 (4)	Reference	
**Parent’s attitude/perception toward influenza vaccine safety in young children**				
Completely safe or very safe	303	44 (15)	2.4 (1.1–5.1)	
Somewhat unsafe, very unsafe or not sure	136	9 (7)	Reference	
**Parent’s agreement with Ministry of Public Health’s recommendation for influenza vaccination in young children**				
Strongly agreed	245	40 (16)	2.7 (1.4–5.2)	2.9 (1.5–5.9)
Agreed, disagreed, strongly disagreed, or not sure	194	13 (7)	Reference	
**Parent ever received influenza vaccine**				
Yes	105	32 (10)	2.4 (1.3–4.3)	
No	334	21 (20)	Reference	
**Child’s age at enrollment (months)**				
6 to <12	139	13 (9)	Reference	
12 to <24	175	25 (14)	1.6 (0.8–3.3)	
24 to <36	125	15 (12)	1.3 (0.6–2.9)	
**Child’s health insurance coverage during the enrollment visit**				
Private insurance or no insurance (paid out-of-pocket)	98	6 (6)	Reference	
Covered by a health insurance provided free of charge by the government or parent’s employer	341	47 (14)	2.6 (1.0–5.9)	2.6 (1.1–6.6)
**Child lived in household where family member(s) smoked**				
Yes	238	22 (9)	0.6 (0.3–1.0)	
No or not sure	201	31 (15)	Reference	
**Child had existing medical condition(s)**^**b**^				
Yes	65	10 (15)	1.4 (0.7–2.9)	
No	374	43 (11)	Reference	
**Child received influenza vaccine in the 2017 influenza season or earlier**				
Yes	29	11 (38)	5.4 (2.4–12.1)	3.2 (1.4–7.8)
No	410	42 (10)	Reference	

95% CI: 95% confidence interval.

^a^The analytic dataset was restricted to include only parents who could make vaccination decisions for their children; all variables associated with vaccination at p-value ≤0.20 in bivariate analyses were entered into multivariable logistic models, the final model was constructed using stepwise backward elimination approach with variables significant at p-value <0.05 retained in the model.

^b^Reported by respondents.

## Discussion

We found that independent determinants of children’s influenza vaccination in 2018 influenza season in Thailand were parents’ considering healthcare providers as a primary source of information about influenza illness, parents’ strongly agreeing with the national recommendation for influenza vaccination in young children, children’s insurance types, and the children’s history of influenza vaccination in the previous season(s).

An agreement with the Thai MOPH’s recommendation for influenza vaccination was a determinant of vaccination in young children in this study as has also been reported in another study conducted in other risk group in Thailand [23]. Not surprisingly, this study along with other studies in China [6, 9], Taiwan [24, 25] and Hong Kong [26] also suggested that children with an experience of influenza vaccination in the past were more likely to receive influenza vaccination in the current year [7–9].

In this study, it is found that while respondents received information from various sources for influenza illness and influenza vaccine, healthcare providers remained the most trusted source of information. Parents who relied on healthcare providers as the information source for influenza illness were nearly three times more likely than those who relied on other sources to have their children vaccinated. This finding is consistent with previous studies in both industrialized and developing countries in Asia [10, 16, 17] and a study conducted in another high-risk group (pregnant women) in Thailand [9]. Additionally, from the various information sources identified, the two most common sources for influenza illness, namely social network and children’s doctors, were cited by nearly 90% of respondents. Similarly, children’s doctors and respondents’ workplace were cited as the two main information outlets for influenza vaccine by nearly 90% of the respondents. This indicates that both social network and workplaces may become promising locations to disseminate health-related information, particularly the MOPH vaccine recommendations.

We found that influenza vaccination coverage in this study was 9% in the 2018 season. The influenza vaccination coverage nationwide in Thai children aged six months to two years was 3% in 2010 [27], 1% in 2012 [27], and 2% in 2015 [4]. The relatively higher vaccination coverage in this study may be due to the local effort to increase influenza vaccination coverage among young children at the network sites before the initiation of this study, and higher media coverage and public awareness regarding influenza vaccination that the MOPH has fostered during recent years. This 9% coverage, however, is lower than the estimated coverage in other high-risk groups in Thailand (34% in elderly [28] and 14% in chronically ill persons [27]) and slightly lower than for children of the same age in other countries in the region e.g., 12% in Hong Kong in 2017 season [11], and 15% in Singapore in 2015 season [15]. This finding underscores the need to strengthen vaccination programs to target specific risk groups with low coverage. In this study, intention to vaccinate the children with influenza vaccine did not always result in children actually being vaccinated possibly because of lack of access to the vaccine or unwillingness to self-purchase the vaccine when free supplies were depleted. Another possible explanation is a desirability bias where the respondents are motivated to answer the survey questions so as to reinforce characteristics and behaviors that are generally socially desirable and deny those that are not.

Some limitations of this study should be acknowledged. First, this study used a cross‑sectional design in which respondents were sampled from hospitals rather than the whole community. Enrollment was also based on convenience sampling; therefore, this sample of children may not represent the entire population of children. Second, the majority of respondents were female (80%) and propensity of gender bias cannot be disregarded while interpreting the results. It is unclear whether there would be any difference in decision-making regarding children’s vaccination against seasonal influenza between fathers and mothers. However, we believe the findings in this study are robust as Thai mothers likely make most vaccination decisions for their children in general.

## Conclusions

The majority of caregivers of children in this study had knowledge of influenza illness and influenza vaccine. Caregivers had diverse socioeconomic and educational backgrounds which influenced their attitude/perception, and practice toward influenza vaccination in the enrolled children. We found that caregivers of vaccinated children were more likely to have higher socio-economic status than those of unvaccinated counterparts. Most caregivers identified healthcare providers as the most trusted source of information about influenza vaccine, but social network and workplace were also identified as important sources of information for many parents. Targeted interventions to disseminate information about influenza illness, influenza vaccine, and the MOPH’s recommendation for vaccination in young children through information sources frequently used by parents may prompt them to talk to their children’s doctors about influenza illness. This, coupled with vaccine distribution policies that produce equitable access to all vaccine target groups, may in turn lead to increase influenza vaccine uptake in children.

## Supporting information

S1 File Questionnaire used to collect knowledge, attitude/perception toward influenza illness and influenza vaccination in the 2018 influenza season in children aged six months to two years. (DOCX)

S2 File Dataset used in the analysis. (XLS)

## List of abbreviations

KAP: Knowledge, Attitude/perception, and Practice
aOR: adjusted odds ratio
CI: confidence interval
MOPH: Ministry of Public Health
