# Supplementary material for: Knowledge, attitude/perception, and practice related to seasonal influenza vaccination among caregivers of young Thai children: A cross-sectional study
Source: PLoS One. 2021 Jun 25;16(6):e0253561. doi: 10.1371/journal.pone.0253561 (PMC8232445; doi:10.1371/journal.pone.0253561)
Supplement: S1 File — (DOCX) [file pone.0253561.s001.docx]

***[English version]***

**Knowledge, Attitudes, and Practices Survey**

Date of interview: ___/___/___

Interviewer’s initials: ___________

Project identification number: __________

**SAY:** These next questions ask your opinions about the flu and seasonal flu vaccination. Seasonal flu vaccination refers to the yearly flu vaccine given to protect against getting the flu that comes around each year.

1. This survey is being done with the child’s

□ 1. Mother

□ 2. Father

□ 3. Another family member (not mother or father)

□ 4. Neighbor or caregiver that is not a family member

□ 5. Other; Specify: __________________

1. How old are you? _____
2. What is the highest level of education that you completed?

□ 1. Never went to school

□ 2. Some primary school

□ 3. Completed primary school

□ 4. Some secondary school

□ 5. Completed secondary school

□ 6. Completed diploma or higher vocational school

□ 7. Completed bachelor’s degree or higher

1. Before your child enrolled in this evaluation, had you heard of the flu (also known as influenza)?

□ 1. Yes

□ 2. No

*STOP, Say: We are finished with the interview. Thank you for your time today.*

□ 3. Unknown

□ 4. Refused

1. How worried are you about your child getting sick with the seasonal flu this year? Please select from the following options that I will read to you. *[Read options 1-4 to the participant before prompting them to answer]*

□ 1. Very worried

□ 2. Somewhat worried

□ 3. Not too worried

□ 4. Not at all worried

□ 5. Unknown

□ 6. Refused

1. If a child who is the same age as your child were to get sick with the seasonal flu, how likely is it that the seasonal flu would harm him or her? Please select from the following options that I will read to you. *[Read options 1-4 to the participant before prompting them to answer]*

□ 1. Very likely

□ 2. Somewhat likely

□ 3. Somewhat unlikely

□ 4. Very unlikely

□ 5. Unknown

□ 6. Refused

1. From which of the following sources had you heard about the flu? Please select from the following options that I will read to you. You may select more than one option. *[Read options 1-16 to the participant]* [Check all that apply]

□ 1. My work

□ 2. My doctor

□ 3. My child’s doctor

□ 4. My child’s school

□ 5. Other medical professionals

□ 6. Other parents

□ 7. Relatives

□ 8. My friends or family members

□ 9. Online websites

□ 10. Community health workers

□11. Television or radio news

□ 12. Newspaper or magazine

□ 13. Social network sites like LINE, Facebook, Twitter, or Instagram

□ 14. Signs or billboards

□ 15. Child’s parent(s)

□ 16. Other source; Specify: ______________

□ 17. Unknown

□ 18. Refused

1. Before your child enrolled in this evaluation, were you aware that there is a vaccine to prevent flu infection in children?

□ 1. Yes, I was aware of the flu vaccine

□ 2. No, I was not aware of the flu vaccine

*Skip to concluding script*

□ 3. Unknown

□ 4. Refused

1. To the best of your knowledge, which of the following *[read options 1 and 2 to the participant before they answer]* do you believe is the current medical recommendation regarding flu vaccination in Thailand for children who are the same age as your child?

□ 1. Children SHOULD get the seasonal flu vaccination each year

□ 2. Children SHOULD NOT get the seasonal flu vaccination each year

□ 3. I don’t know the current recommendation

□ 4. Refused

1. From which of the following sources had you heard about the flu vaccine for children? Please select from the following options that I will read to you. You may choose more than one. *[Read options 1-16 to the participant]* [Check all that apply]

□ 1. My work

□ 2. My doctor

□ 3. My child’s doctor

□ 4. My child’s school

□ 5. Other medical professionals

□ 6. Other parents

□ 7. Relatives

□ 8. My friends or family members

□ 9. Online websites

□ 10. Community health workers

□ 11. Television or radio news

□ 12. Newspaper or magazine

□ 13. Social network sites like LINE, Facebook, Twitter, or Instagram

□ 14. Signs or billboards

□ 15. Child’s parent(s)

□ 16. Other source; Specify: ______________

□ 17. Unknown

□ 18. Refused

1. How well do you think the flu vaccine works in protecting children who are the same age as your child from getting the flu? Please select from the following options that I will read to you. *[Read options 1-4 to the participant before prompting them to answer]*

□ 1. Very well

□ 2. Somewhat well

□ 3. Not too well

□ 4. Not at all

□ 5. Unknown

□ 6. Refused

1. Based on what you know or have heard, how safe are seasonal flu vaccines for children? Please select from the following options that I will read to you. *[Read options 1-6 to the participant before prompting them to answer]*

□ 1. Completely safe

□ 2. Very safe

□ 3. Somewhat safe

□ 4. Somewhat unsafe

□ 5. Very unsafe

□ 6. Completely unsafe

□ 7. Unknown

□ 8. Refused

1. Please indicate how much you agree or disagree with the following statement: children should get the seasonal flu vaccine. Please select from the following options that I will read to you. *[Read options 1-4 to the participant before prompting them to answer]*

□ 1. Strongly agree

□ 2. Agree

□ 3. Disagree

□ 4. Strongly disagree

□ 5. Unknown

□ 6. Refused

1. Which ONE of these sources do you trust the most for receiving information about seasonal flu vaccine? Please select only ONE from the following options that I will read to you. *[Read options 1-16 to the participant]* [Choose one source only]

□ 1. My work

□ 2. My doctor

□ 3. My child’s doctor

□ 4. My child’s school

□ 5. Other medical professionals

□ 6. Other parents

□ 7. Relatives

□ 8. My friends or family members

□ 9. Online websites

□ 10. Community health workers

□ 11. Television or radio news

□ 12. Newspaper or magazine

□ 13. Social network sites like LINE, Facebook, Twitter, or Instagram

□ 14. Signs or billboards

□ 15. Child’s parent(s)

□ 16. Other source; Specify: ______________

□ 17. Unknown

□ 18. Refused

1. *[Check response to question #8 on Form F: Enrollment Interview. If the answer is “No” or “Do not know”, proceed with this question. If the answer is “Yes”, skip to question 16]* If you were offered flu vaccine for your child this year, would you have your child vaccinated?

□ 1. Yes

□ 2. No

□ 3. Unknown

□ 4. Refused

1. Have you ever had a seasonal flu vaccine?

□ 1. Yes

□ 2. No

□ 3. Unknown

□ 4. Refused

**SAY:** We are finished with the interview. Thank you for your time today.

Do you have any questions for me? *[answer any questions]*

# *[Thai version]*

# แบบสัมภาษณ์เกี่ยวกับความรู้ ทัศนคติและการปฏิบัติตัว

**วันที่สัมภาษณ์**: ___/___/___

ชื่อย่อของเจ้าหน้าที่โครงการ: ___ ___

*หมายเลขประจำตัวอาสาสมัคร*: ___________

**กล่าวกับพ่อแม่/ผู้ดูแลอาสาสมัคร:** “คำถามต่อไปนี้จะถามความเห็นของคุณเกี่ยวกับโรคไข้หวัดใหญ่และวัคซีนป้องกันไข้หวัดใหญ่ประจำฤดูกาล วัคซีนป้องกันไข้หวัดใหญ่ประจำฤดูกาลหมายถึงวัคซีนป้องกันไข้หวัดใหญ่ที่ต้องฉีดทุกปี เพื่อป้องกันการป่วยด้วยโรคไข้หวัดใหญ่ในแต่ละปี”

1. ผู้ที่ตอบแบบสอบถามชุดนี้เกี่ยวข้องอะไรกับอาสาสมัคร

□ 1. แม่

□ 2. พ่อ

□ 3. สมาชิกในครอบครัวคนอื่นๆ (แต่ไม่ใช่แม่หรือพ่อ)

□ 4. เพื่อนบ้านหรือผู้ดูแลอาสาสมัครซึ่งไม่ใช่สมาชิกในครอบครัว

□ 5. อื่นๆ; ระบุ: __________________

1. คุณอายุเท่าไหร่? _____
2. คุณจบการศึกษาขั้นสูงสุดชั้นใด?

□ 1. ไม่เคยเข้าเรียนในโรงเรียน

□ 2. ประถมศึกษาแต่ไม่จบ

□ 3. จบประถมศึกษา

□ 4. มัธยมศึกษาแต่ไม่จบ

□ 5. จบมัธยมศึกษา

□ 6. จบระดับประกาศนียบัตรหรือปวส.

□ 7. จบปริญญาตรีหรือสูงกว่า

1. ก่อนที่บุตร/เด็กในความดูแลของคุณจะเข้าร่วมโครงการนี้ คุณเคยได้ยินเกี่ยวกับโรคไข้หวัดใหญ่บ้างหรือไม่?

□ 1. เคย

*ยุติ กล่าวกับพ่อแม่/ผู้ดูแล:* “*จบการสัมภาษณ์แล้ว โครงการขอขอบคุณที่คุณสละเวลาให้สัมภาษณ์ในวันนี้*”

□ 2. ไม่เคย

□ 3. ไม่ทราบ

□ 4. ไม่ตอบ

1. คุณมีความกังวลมากน้อยเพียงใดในการที่บุตร/เด็กความดูแลจะป่วยด้วยโรคไข้หวัดใหญ่ในปีนี้? กรุณาเลือกคำตอบจากตัวเลือกต่อไปนี้ที่ดิฉัน/ผมจะอ่านให้ฟัง *[*อ่านเฉพาะตัวเลือกที่ 1-4 แล้วรอฟังคำตอบ*]*

□ 1. กังวลมาก

□ 2. กังวลบ้าง

□ 3. ไม่กังวลมากนัก

□ 4. ไม่กังวล

□ 5. ไม่ทราบ

□ 6. ไม่ตอบ

1. หากมีเด็กที่อายุใกล้เคียงกับบุตร/เด็กในความดูแลของคุณเกิดป่วยด้วยโรคไข้หวัดใหญ่ตามฤดูกาลขึ้นมา คุณคิดว่าเด็กได้รับอันตรายมากน้อยเพียงใดจากการป่วยนี้? กรุณาเลือกคำตอบจากตัวเลือกต่อไปนี้ที่ดิฉัน/ผมจะอ่านให้ฟัง *[*อ่านเฉพาะตัวเลือกที่ *1-4* แล้วรอฟังคำตอบ]

□ 1. มากที่สุด

□ 2. มาก

□ 3. ไม่น่าจะเป็นอันตราย

□ 4. ไม่เป็นอันตราย

□ 5. ไม่ทราบ

□ 6. ไม่ตอบ

1. คุณเคยได้ยินเรื่องไข้หวัดใหญ่จากที่ใดบ้าง? กรุณาเลือกคำตอบจากตัวเลือกต่อไปนี้ที่ดิฉัน/ผมจะอ่านให้ฟัง คุณสามารถตอบได้มากกว่าหนึ่งข้อ *[*อ่านเฉพาะตัวเลือกที่ *1-16* ให้อาสาสมัครฟัง] [เลือกคำตอบทั้งหมดที่อาสาสมัครตอบ]

□ 1. ที่ทำงานของคุณ

□ 2. แพทย์ประจำตัวของคุณ

□ 3. แพทย์ของบุตร/เด็กในความดูแล

□ 4. โรงเรียนของบุตร/เด็กในความดูแล

□ 5. สมาคมหรือชมรมทางด้านการแพทย์ต่างๆ

□ 6. พ่อแม่คนอื่นๆ

□ 7. ญาติ

□ 8. เพื่อนหรือคนในครอบครัว

□ 9. เวปไซด์

□ 10. เจ้าหน้าที่สาธารณสุขในชุมชน

□ 11. โทรทัศน์หรือวิทยุ

□ 12. หนังสือพิมพ์หรือนิตยสาร

□ 13. สังคมออนไลน์ เช่น ไลน์ เฟสบุค ทวิตเตอร์ หรืออินสตราแกรม

□ 14. ป้ายต่างๆ

□ 15. พ่อ/แม่ของเด็กในความดูแล

□ 16. อื่นๆ; ระบุ: ______________

□ 17. ไม่ทราบ

□ 18. ไม่ตอบ

1. ก่อนที่บุตร/เด็กในความดูแลจะเข้าร่วมโครงการนี้ คุณเคยทราบเกี่ยวกับวัคซีนป้องกันไข้หวัดใหญ่ที่สามารถป้องกันเด็กจากการเป็นไข้หวัดใหญ่หรือไม่?

□ 1. ทราบเกี่ยวกับวัคซีน

□ 2. ไม่ทราบเกี่ยวกับวัคซีน

*ข้ามไปที่บทพูดสิ้นสุดการสัมภาษณ์*

□ 3. ไม่แน่ใจ

□ 4. ไม่ตอบ

1. เท่าที่คุณทราบ ข้อใดต่อไปนี้เป็นข้อที่ถูกต้องเกี่ยวกับคำแนะนำในประเทศไทยในปัจจุบันเรื่องการฉีดวัคซีนป้องกันไข้หวัดใหญ่แก่เด็กที่มีอายุเท่ากับบุตร/เด็กในความดูแลของคุณ? *[*อ่านตัวเลือกข้อ *1* และ 2 ให้อาสาสมัครฟัง*]*

□ 1. เด็กควรได้รับการฉีดวัคซีนป้องกันไข้หวัดใหญ่ประจำฤดูกาลทุกปี

□ 2. เด็กไม่ควรได้รับการฉีดวัคซีนป้องกันไข้หวัดใหญ่ประจำฤดูกาลทุกปี

□ 3. ไม่ทราบเกี่ยวกับคำแนะนำ

□ 4. ไม่ตอบ

1. คุณเคยได้ยินเรื่องวัคซีนป้องกันไข้หวัดใหญ่จากแหล่งใดบ้าง? กรุณาเลือกคำตอบจากตัวเลือกต่อไปนี้ที่ดิฉัน/ผมจะอ่านให้ฟัง คุณสามารถเลือกได้มากกว่าหนึ่งคำตอบ *[*อ่านตัวเลือกข้อที่ *1-16* ให้อาสาสมัครฟัง*]* [เลือกคำตอบทั้งหมดที่อาสาสมัครตอบ]

□ 1. ที่ทำงานของคุณ

□ 2. แพทย์ประจำตัวของคุณ

□ 3. แพทย์ของบุตร/เด็กในความดูแล

□ 4. โรงเรียนของบุตร/เด็กในความดูแล

□ 5. สมาคมหรือชมรมทางด้านการแพทย์ต่างๆ

□ 6. พ่อแม่คนอื่นๆ

□ 7. ญาติ

□ 8. เพื่อนหรือคนในครอบครัว

□ 9. เวปไซด์

□ 10. เจ้าหน้าที่สาธารณสุขในชุมชน

□ 11. โทรทัศน์หรือวิทยุ

□ 12. หนังสือพิมพ์หรือนิตยสาร

□ 13. สังคมออนไลน์ เช่น ไลน์ เฟสบุค ทวิตเตอร์ หรืออินสตราแกรม

□ 14. ป้ายต่างๆ

□ 15. พ่อ/แม่ของเด็กในความดูแล

□ 16. อื่นๆ; ระบุ: ______________

□ 17. ไม่ทราบ

□ 18. ไม่ตอบ

1. คุณคิดว่าวัคซีนป้องกันไข้หวัดใหญ่สามารถป้องกันโรคไข้หวัดใหญ่ในเด็กอายุเท่ากับบุตร/เด็กในความดูแลของคุณได้ดีเพียงใด? กรุณาเลือกคำตอบจากตัวเลือกต่อไปนี้ที่ดิฉัน/ผมจะอ่านให้ฟัง *[*อ่านเฉพาะตัวเลือกที่ 1-4 แล้วรอฟังคำตอบ]

□ 1. ป้องกันได้ดีมาก

□ 2. ป้องกันได้ดีพอควร

□ 3. ป้องกันได้ไม่ดีมากนัก

□ 4. ไม่ป้องกันเลย

□ 5. ไม่ทราบ

□ 6. ไม่ตอบ

1. จากที่คุณได้ทราบหรือได้ยินมา คุณคิดว่าวัคซีนป้องกันไข้หวัดใหญ่ประจำฤดูกาลมีความปลอดภัยมากน้อยเพียงใดในเด็ก? กรุณาเลือกคำตอบจากตัวเลือกต่อไปนี้ที่ดิฉัน/ผมจะอ่านให้ฟัง *[*อ่านเฉพาะตัวเลือกที่ *1-6* แล้วรอฟังคำตอบ]

□ 1. ปลอดภัยมากที่สุด

□ 2. ปลอดภัยมาก

□ 3. ค่อนข้างปลอดภัย

□ 4. ไม่ค่อยปลอดภัย

□ 5. ไม่ปลอดภัยเลย

□ 6. ไม่ปลอดภัยมากที่สุด

□ 7. ไม่ทราบ

□ 8. ไม่ตอบ

1. กรุณาเลือกตอบว่าคุณเห็นด้วยหรือไม่เห็นด้วยกับประโยคต่อไปนี้: เด็กควรได้รับวัคซีนป้องกันไข้หวัดใหญ่ประจำฤดูกาล กรุณาเลือกคำตอบจากตัวเลือกต่อไปนี้ที่ดิฉัน/ผมจะอ่านให้ฟัง *[*อ่านเฉพาะตัวเลือกที่ *1-4* แล้วรอฟังคำตอบ]

□ 1. เห็นด้วยมากที่สุด

□ 2. เห็นด้วย

□ 3. ไม่เห็นด้วย

□ 4. ไม่เห็นด้วยมากที่สุด

□ 5. ไม่ทราบ

□ 6. ไม่ตอบ

1. ข้อใดต่อไปนี้เป็นแหล่งข้อมูลที่คุณเชื่อมากที่สุดในการรับข้อมูลเกี่ยวกับวัคซีนป้องกันไข้หวัดใหญ่ประจำฤดูกาล? กรุณาเลือกตอบเพียงข้อเดียวจากตัวเลือกที่ดิฉัน/ผมจะอ่านให้ฟัง *[*อ่านตัวเลือกที่ *1-16* ให้อาสาสมัครฟัง*]* [เลือกได้เพียงหนึ่งคำตอบ]

□ 1. ที่ทำงานของคุณ

□ 2. แพทย์ประจำตัวของคุณ

□ 3. แพทย์ของบุตร/เด็กในความดูแล

□ 4. โรงเรียนของบุตร/เด็กในความดูแล

□ 5. สมาคมหรือชมรมทางด้านการแพทย์ต่างๆ

□ 6. พ่อแม่คนอื่นๆ

□ 7. ญาติ

□ 8. เพื่อนหรือคนในครอบครัว

□ 9. เวปไซด์

□ 10. เจ้าหน้าที่สาธารณสุขในชุมชน

□ 11. โทรทัศน์หรือวิทยุ

□ 12. หนังสือพิมพ์หรือนิตยสาร

□ 13. สังคมออนไลน์ เช่น ไลน์ เฟสบุค ทวิตเตอร์ หรืออินสตราแกรม

□ 14. ป้ายต่างๆ

□ 15. พ่อ/แม่ของเด็กในความดูแล

□ 16. อื่นๆ; ระบุ: ______________

□ 17. ไม่ทราบ

□ 18. ไม่ตอบ

1. *[ถามคำถามข้อนี้ หากอาสาสมัครตอบคำถามข้อ 8 ในแบบบันทึก F: แบบสัมภาษณ์แรกเข้าโครงการการว่า “ไม่ได้รับการฉีดวัคซีน” หรือ “ไม่ทราบ/ไม่แน่ใจ” หากตอบว่า “ได้รับการฉีดวัคซีน” ข้ามไปคำถามข้อ 16]* หากบุตร/เด็กในความดูแลของคุณได้รับการเสนอให้ฉีดวัคซีนป้องกันไข้หวัดใหญ่ในปีนี้ คุณจะอนุญาตให้เด็กฉีดวัคซีนหรือไม่?

□ 1. อนุญาต

□ 2. ไม่อนุญาต

□ 3. ไม่ทราบ

□ 4. ไม่ตอบ

1. ตัวคุณเองเคยฉีดวัคซีนป้องกันไข้หวัดใหญ่หรือไม่?

□ 1. เคย

□ 2. ไม่เคย

□ 3. ไม่ทราบ

□ 4. ไม่ตอบ

**กล่าวแก่อาสาสมัคร**: **“ตอนนี้หมดคำถามแล้ว โครงการขอขอบคุณที่คุณได้สละเวลาให้สัมภาษณ์ในวันนี้”**

คุณมีคำถามใดๆ ไหมครับ/ค่ะ? *[ตอบคำถามที่อาสาสมัครถาม]*
